# Antimicrobial Resistance and Selected Virulence-Associated Genes *Escherichia coli* Pathotypes in Free-Living Cats from Southern Spain

**DOI:** 10.3390/ani16152435

**Published:** 2026-08-06

**Authors:** Gómez-Gascón Lidia, Romero-Salmoral Antonio, Huerta Lorenzo Belén, Galán-Relaño Ángela, Marco-Fuertes Ana, Mena-Rodríguez Mª Ángeles, Molina Guillén Eva, Rafael J. Astorga Márquez

**Affiliations:** 1 Departamento de Sanidad Animal, Facultad de Veterinaria, Universidad de Cordoba, 14014 Córdoba, Spain; v32gogal@uco.es (G.-G.L.); sa2hulob@uco.es (H.L.B.); mariangii53@gmail.com (M.-R.M.Á.); sa1asmar@uco.es (R.J.A.M.); 2 Unidad de Investigación Competitiva, Zoonosis y Enfermedades Emergentes (ENZOEM), Universidad de Cordoba, 14014 Córdoba, Spain; 3 Departamento de Enfermería, Farmacología y Fisioterapia, Área de Farmacología, Facultad de Veterinaria, Universidad de Cordoba, 14014 Córdoba, Spain; agalanr12@gmail.com; 4 Facultad de Veterinaria, Instituto de Ciencias Biomédicas, Universidad Cardenal Herrera-CEU, CEU Universities, 46115 Valencia, Spain; ana.marcofuertes@uchceu.es; 5 Centro Veterinario Fénix, 29639 Benalmadena, Spain; evillamolinag@gmail.com

**Keywords:** stray cats, *Escherichia coli*, antimicrobial resistance, multidrug resistance, atypical enteropathogenic *E. coli* (aEPEC), One Health

## Abstract

Stray cats may act as reservoirs of antimicrobial-resistant and potentially pathogenic bacteria, representing a potential public health concern within a One Health framework. This study investigated the occurrence of antimicrobial resistance (AMR), multidrug resistance (MDR), and virulence-associated traits in commensal *Escherichia coli* isolated from free-living cat colonies in southern Spain. A total of 169 rectal swabs were collected from cats belonging to feline colonies and shelters in Benalmádena (Málaga, Spain). Bacterial isolation and identification were performed using selective culture media, conventional biochemical tests, and MALDI-TOF mass spectrometry. Antimicrobial susceptibility was determined by minimum inhibitory concentration (MIC) testing against 15 antimicrobial agents, and isolates were screened for selected virulence-associated genes associated with major diarrheagenic *E. coli* pathotypes. A total of 68 *E. coli* isolates (40.2%) were recovered. The highest resistance frequencies were observed for sulfamethoxazole (25.0%) and ampicillin (20.6%), whereas all isolates showed high susceptibility rates to most of the antimicrobials tested, including azithromycin (100%), as well as gentamicin, amikacin, cefotaxime, ceftazidime, meropenem, colistin, chloramphenicol and tigecycline (98.5%). Six isolates (8.8%) were classified as multidrug-resistant. In addition, six isolates (8.8%) were identified as atypical enteropathogenic *E. coli* (aEPEC), although none exhibited a multidrug resistance phenotype. These findings demonstrate the presence of both AMR and virulence-associated traits among commensal *E. coli* circulating in free-living cats. The inclusion of feline colonies or shelters in AMR surveillance programmes may contribute valuable information for integrated One Health monitoring strategies.

## Simple Summary

Antimicrobial resistance (AMR) is a major global health challenge that requires a One Health approach integrating human, animal, and environmental health. Free-living cats may contribute to the maintenance and dissemination of antimicrobial-resistant and potentially pathogenic bacteria because they share urban environments with humans, domestic animals, and wildlife. In this study, commensal *Escherichia coli* isolates recovered from free-living cat colonies in southern Spain were characterized for AMR and selected virulence-associated genes. A total of 68 *E. coli* isolates were recovered from 169 rectal swabs. Resistance was most frequently observed to sulfamethoxazole (25.0%) and ampicillin (20.6%), while six isolates (8.8%) were classified as multidrug-resistant (MDR). In addition, six isolates (8.8%) were identified as atypical enteropathogenic *E. coli* (aEPEC). These findings highlight the importance of including free-living cats in AMR surveillance programmes and support the implementation of integrated One Health monitoring strategies.

## 1. Introduction

The emergence and spread of antimicrobial resistance (AMR) represent a global health problem that requires a One Health approach [1]. In Europe, veterinary antimicrobial consumption was monitored by the ‘European Surveillance of Veterinary Antimicrobial Consumption (ESVAC)’ project from 2009 to 2023 [2]. Currently, in response to European regulations, a modified version of this program has been implemented: the ‘European Sales and Use of Antimicrobials for Veterinary Medicine (ESUAvet)’, as published in European Medicines Agency (EMA) webpage. This program developed a harmonized approach for collecting and reporting data on the sale and use of antibiotics in animals. However, the monitoring of AMR among animals is not systematic across European countries, as it focuses mainly on food-producing animals, and not on companion animals such as dogs and cats [3,4]. In Spain, the ‘Antimicrobial Resistance National Program’ (PRAN) has been in force since 2014, and for the period 2025–2027, the PRAN intends to include AMR in companion animals within the ‘reduce antibiotics program’ [5].

Pet animals, particularly cats and dogs, are increasingly recognized as potential reservoirs for the transmission of AMR/Multidrug resistance (MDR) to humans. This concern arises from two key factors: the frequent and sometimes inappropriate use of broad-spectrum antimicrobial agents in veterinary medicine, and the close, often daily, physical contact between companion animals and their human owners. Such proximity facilitates not only microbial exchange but also the potential for resistant isolates to spread across species [5]. Several studies have demonstrated the presence of multidrug-resistant bacteria in clinically healthy pets, indicating that even asymptomatic animals can harbour and disseminate resistant microorganisms. As highlighted by Li et al. [6], AMR has been detected in bacterial isolates obtained from both cats and dogs across the Iberian Peninsula, raising significant public health concerns.

Transmission of AMR bacteria between companion animals and humans, particularly cats, may occur through direct or indirect contact and represents a potential public health concern [7]. Despite all this, it is important to note that the risk of contracting an infection from a veterinarian-supervised domesticated cat is low; however, this risk increases substantially when in contact with stray cats [7]. However, stray cats that are in contact with humans and share the urban environment with them may act as reservoirs and disseminators of antimicrobial-resistant bacteria affecting humans and companion animals.

Free-living cats that live outdoors in public or private urban areas, usually live in colonies, using human activity resources to feed or prey on a broad biodiversity of animals such as birds, rodents, and reptiles [8,9]. Populations of free-living cats could represent an important threat to public health, being a significant factor in the transmission of zoonotic diseases due to their close contact with humans and other pets [10,11]. In fact, previous studies, carried out in different countries, have reported cats as a source of zoonotic infectious and parasitic diseases [8,12,13,14].

In addition to AMR, *Escherichia coli* (*E. coli*) isolated from companion animals may harbour virulence factors associated with different diarrheagenic pathotypes, including enteropathogenic (EPEC), Shiga toxin-producing (STEC), enterotoxigenic (ETEC), enteroaggregative (EAEC), and enteroinvasive (EIEC) *E. coli* [15]. Among these, EPEC is responsible for millions of diarrhoeal cases worldwide and represents an important public health concern [16]. EPEC isolates are classified as typical (tEPEC) or atypical (aEPEC) according to the presence or absence of the *EAF* plasmid carrying the *bundle-forming pilus* gene (*bfpA*), while both groups possess the *eae* gene encoding intimin [16,17,18]. Cats have been described as asymptomatic carriers of diarrheagenic *E. coli* pathotypes, including aEPEC, highlighting their potential role in the maintenance and dissemination of virulence-associated genes.

In European countries, the number of studies focusing on cats is still very limited. This may be due to a lower interest in this species compared to dogs, but also to logistical challenges such as difficulty in handling and sampling these animals or obtaining faecal samples [19,20]. Nonetheless, different publications have reviewed the presence of zoonotic pathogens in feline colonies in Europe. For example, Rosario et al. [21] described for the first time the presence of *Salmonella enterica* subsp. *enterica* in feline colonies in Las Palmas de Gran Canaria (Canary Island, Spain), assessing its impact on public health. Additionally, several studies in Italy, using a strong One Health approach, have specifically investigated the presence of *E. coli* in faecal samples from feline colonies, as well as its AMR phenotype. Thus, Ratti et al. [22] and Gargano et al. [23] confirmed, respectively, the circulation of multidrug-resistant *E. coli* isolates in feral cats, including isolates producing ESBL (Extended Spectrum β-Lactamases).

Although several studies in Spain have reported the role of companion animals as reservoirs of AMR [6,24,25], limited information is available regarding the role and circulation of *E. coli* and AMR-linked isolates in free-living cats in our country. In this sense, few studies have focused on the role of stray cats as disseminators of resistance to broad-spectrum antibiotics used in veterinary and human medicine [26].

Considering this, the present study aimed to investigate AMR, MDR, and virulence-associated traits in commensal *E. coli* isolated from fecal samples collected from free-living cats. To achieve this, antimicrobial susceptibility was assessed phenotypically, and the isolates were screened for selected virulence-associated genes related to major diarrheagenic *E. coli* pathotypes.

## 2. Material and Methods

### 2.1. Sample Collection

This collaborative research work was carried out between the Epidemiology, Preventive Medicine, and Health Policy Unit of the Animal Health Department (Veterinary Faculty, University of Córdoba, Spain), the Health and Environment area of the Benalmádena City Council (Málaga province), and the Fénix veterinary clinic located in Benalmádena (Málaga province), a ‘Cat Friendly Silver Level Centre’ Categorized by the International Society of Feline Medicine (ISFM).

**Figure 1:**
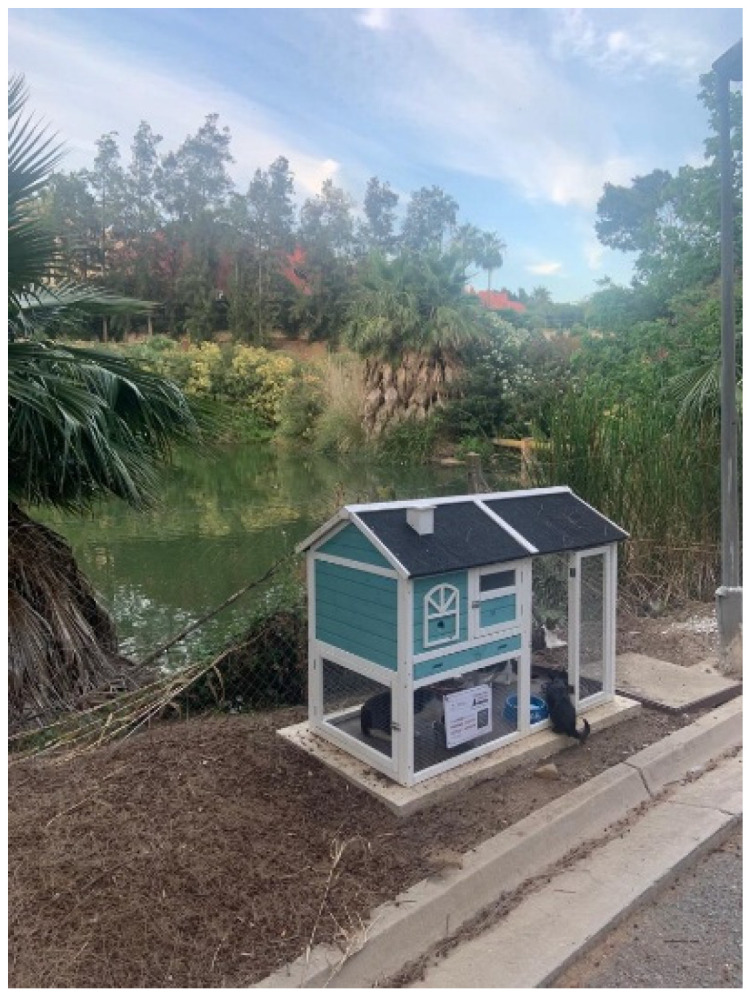
Shelter for feral cats (Benalmadena, Province of Málaga).

The study population consisted of all free-living and shelter cats from Benalmádena that were admitted to the Fénix Veterinary Clinic for routine surgical neutering during the study period as part of the official Trap–Neuter–Return (TNR) programme or shelter management. Rectal swabs were collected from every cat before surgery; therefore, no random sampling was performed. In addition, neutered free-living cats were permanently identified by ear-tipping, following standard TNR practice, which prevented duplicate sampling during subsequent capture campaigns. A total of 169 sterile rectal swabs were obtained from cats belonging to free-living colonies or shelters in Benalmádena (Málaga, Spain) between October 2022 and March 2023. Rectal swabs were collected from cats originating from free-living colonies and a local shelter in Benalmádena (Málaga, Spain) between October 2022 and March 2023. Feline colonies are large groups of free-living cats, mainly inhabiting urban areas and originating from abandoned or stray animals and their offspring, whereas shelters house cats in a semi-domestic environment with appropriate sanitary measures (e.g., sterilization and deworming) and adoption as their primary goal. All samples were collected at the Fénix Veterinary Clinic during routine surgical neutering procedures within the framework of the official Trap–Neuter–Return (TNR) programme implemented by the Benalmádena City Council. Rectal swabs were collected by a licensed veterinarian under aseptic conditions using sterile, individually packaged swabs while the animals were under general anaesthesia. Care was taken to avoid contact of the swab with any non-target surfaces before rectal insertion. Each animal was sampled only once. Following collection, each swab was immediately placed into sterile Amies transport medium and transported at ≤4 °C to the laboratory of the Animal Health Department (University of Córdoba) for microbiological analyses within 24 h (Figure 1).

### 2.2. E. coli Isolates Identification

Rectal swabs were pre-enriched in buffered peptone water (BPW; Scharlau, Barcelona, Spain), in 1:10 *v*/*v* proportion, and incubated at 37 ± 1 °C for 24 ± 2 h. All the pre-enriched samples were inoculated onto XLD (Xylose Lysine Deoxycholate) agar medium and incubated for 24 h at 37 °C. Colonies showing morphological characteristics compatible with *E. coli* (yellow, smooth, round, and convex colonies on XLD agar) were selected and subcultured as necessary to obtain pure cultures.

Only one isolate per animal was subcultured on brain–heart infusion agar incubated at 37 °C for 24 h and confirmed as *E. coli* by biochemical tests including: Gram stain, catalase test, oxidase test and indole production test, before species confirmation by Matrix-Assisted Laser Desorption–Ionization Time-of-Flight Mass Spectrometry (MALDI-TOF) (Bruker MALDI Biotyper, Bremen, Germany).

### 2.3. Antimicrobial Susceptibility Testing

Antimicrobial susceptibility testing was performed using the Minimum Inhibitory Concentration (MIC) assay with the *Salmonella*/*E. coli* EUVSEC Plate (Thermo Scientific, Sensititre Plate Guide, Madrid, Spain), which includes the antimicrobial agents required by Decision (EU) 2020/1729 for monitoring AMR in zoonotic and commensal bacteria. The following antimicrobial agents were tested according to their respective classes and concentration ranges (µg/mL): aminopenicillins (ampicillin, AMP, 1–32), third-generation cephalosporins (cefotaxime, CTX, 0.25–8; ceftazidime, CAZ, 0.5–16), carbapenems (meropenem, MEM, 0.03–4), quinolones (nalidixic acid, NA, 4–64; ciprofloxacin, CIP, 0.015–4), macrolides (azithromycin, AZM, 2–64), aminoglycosides (gentamicin, GEN, 0.25–16; amikacin, AMK, 2–64), polymyxins (colistin, CL, 0.25–4), sulphonamides (sulfamethoxazole, SF, 16–512), diaminopyrimidines (trimethoprim, TMP, 0.25–8), amphenicols (chloramphenicol, CHL, 2–32), tetracyclines (tetracycline, TE, 1–16), and glycylcyclines (tigecycline, TGC, 0.015–2). The results were interpreted using the Sensititre™ SWIN™ Software V3.4 System based on clinical breakpoints from CLSI M100 and EUCAST [27]. Antimicrobial susceptibility results were interpreted according to EUCAST clinical breakpoints for *Enterobacterales* whenever available. For nalidixic acid and tetracycline, CLSI breakpoints were applied due to the absence of corresponding EUCAST clinical breakpoints. For azithromycin and chloramphenicol, clinical breakpoints were not available for *E. coli* in either EUCAST or CLSI; therefore, epidemiological cut-off values (ECOFFs) were used to distinguish wild-type (WT) isolates from isolates with acquired resistance mechanisms (non-WT). For statistical analyses, resistant and non-wild-type (non-WT) isolates were grouped into a single category and compared with susceptible and wild-type (WT) isolates.

Isolates showing acquired resistance to at least one antimicrobial agent in three or more antimicrobial classes were classified as MDR [28].

According to the EMA classification for the prudent and responsible use of antibiotics in animals [29], the antimicrobials included in this study were categorized into four groups: Category D (“Prudence”), considered first-line therapeutic options; Category C (“Caution”), recommended when Category D antimicrobials are not effective; Category B (“Restrict”), corresponding to the Highest Priority Critically Important Antimicrobials (HPCIAs) included in the World Health Organization (WHO) list of Medically Important Antimicrobials (MIAs), including third- and fourth-generation cephalosporins, quinolones, polymyxins, and phosphonic acid derivatives; and Category A (“Avoid”), comprising antimicrobials not authorized for veterinary use and considered last-resort drugs in human medicine [30]. Within the European Union, these antimicrobials are reserved for human use and are not authorized for food-producing animals, although they may be prescribed under exceptional circumstances in companion animals in accordance with current regulations [29].

### 2.4. Genotypic Characterization

Crude DNA extracts from bacterial isolates were used as DNA templates for PCR assays. Briefly, colonies on agar were picked and suspended in 300 μL of ultrapure water, boiled for 10 min, centrifuged at 13,000 revolutions per minute (rpm) for 30 s and adjusted to a DNA concentration of 10–20 ng/µL using a NanoDrop 2000/2000C spectrophotometer (Thermo Fisher Scientific^TM^, Waltham, MA, USA).

Because azithromycin susceptibility is not routinely included in most AMR surveillance studies of commensal *E. coli* and its interpretation relies on epidemiological cut-off values (ECOFFs), a complementary PCR screening targeting selected macrolide resistance genes was performed to support the phenotypic findings. No additional resistance determinants were investigated, as the primary objective of the study was the phenotypic characterization of antimicrobial susceptibility.

Amplification was carried out using previously described primers targeting a panel of 10 acquired macrolide resistance genes and three chromosomal loci associated with macrolide resistance [31]. Primer sequences, target genes, expected amplicon sizes, and PCR conditions are provided in Table S1.

To determine the presence of diarrheagenic *E. coli* pathotypes, conventional PCR was performed using six pairs of specific primers (Supplementary Table S2) targeting different virulence-associated genes as previously described [15]. Each PCR assay contained a final reaction volume of 24 µL, consisting of 12 µL of Qiagen PCR Master Mix (Qiagen, Hilden, Germany), 0.5 µL of each primer (20 µM stock concentration; final concentration 0.42 µM), 9 µL of nuclease-free water, and 2 µL of DNA template. All PCR reactions were performed in duplicate. Amplification was performed using a cycling protocol with different annealing temperatures depending on each pair of primers: an initial denaturation at 95 °C for 3 min; followed by 35 cycles at 95 °C for 30 s, primer-specific annealing temperature (Supplementary Table S2), and 72 °C for 40 s. A final extension step was performed at 72 °C for 10 min, followed by maintenance at 12 °C.

Positive controls carrying the corresponding target genes and a negative control were included in each PCR run.

Amplified products were separated by electrophoresis on 2% (*w*/*v*) agarose gels. Samples were mixed with loading buffer containing bromophenol blue prior to loading. DNA bands were visualized under UV illumination following SYBR Safe staining.

Isolates were classified into the corresponding pathotypes according to the virulence gene profiles detected by PCR, following previously established molecular criteria for pathogenic *E. coli* characterization [15].

### 2.5. Statistical Analysis

The Multiple Antimicrobial Resistance index (MAR index) value [32] was calculated for each isolate according to Matos et al. [33] as the ratio between the number of antimicrobials to which the isolate was resistant and the total number of antimicrobials tested. The mean MAR index was subsequently calculated for the study population. MAR values <0.2 were considered indicative of isolates originating from environments with low antimicrobial exposure, whereas MAR values ≥ 0.2 suggested exposure to environments with frequent antimicrobial use [34].

On the other hand, associations between resistance phenotypes were evaluated using Spearman’s rank correlation coefficient (PROC CORR). For this analysis, antimicrobial susceptibility results were categorized as susceptible or resistant, with intermediate isolates considered resistant [34].

Statistical significance of Spearman’s rank correlation coefficients was established at *p* < 0.05.

## 3. Results

### 3.1. E. coli Isolates Identification

From 169 rectal swabs collected from cats housed in free-living colonies and shelters, a total of 68 commensal *E. coli* isolates (40.23%) were identified using conventional biochemical tests and subsequently confirmed by MALDI-TOF mass spectrometry. Only one presumptive *E. coli* colony was selected per sample, ensuring that each isolate corresponded to a different animal. These isolates were stored at −20 °C in brain–heart infusion broth with 15% glycerol until MIC analysis.

### 3.2. Genotypic Characterization

Among the 68 commensal *E. coli* isolates analysed, 6 (8.8%) carried the *eae* (intimin) virulence gene. According to their virulence gene profile, these isolates were identified as atypical enteropathogenic *E. coli* (aEPEC), since they were positive for *eae* but negative for *bfpA*. No additional virulence genes included in the study were detected among the analysed isolates (Table 1). In addition, none of the isolates carried the macrolide resistance genes investigated.

### 3.3. Antimicrobial Susceptibility Testing

*E. coli* isolates showed high susceptibility rates to most of the antimicrobials tested, including azithromycin (100%), as well as gentamicin, amikacin, cefotaxime, ceftazidime, meropenem, colistin, chloramphenicol and tigecycline (98.5%) (Table 2).

The highest frequencies of resistance were observed for sulphonamides (25%) and ampicillin (20.6%), both included in EMA Category D (“Prudence”). Lower resistance rates were detected for nalidixic acid, tetracycline and trimethoprim (7.4% each), and ciprofloxacin (5.9%) (Table 2). Meropenem and tigecycline belong to Category A (“Avoid”) according to the EMA categorization and are considered CIA reserved for human medicine. Resistance to these agents was absent or very low, with resistance rates of 1.5% for tigecycline and meropenem (Table 2).

**Figure 2:**
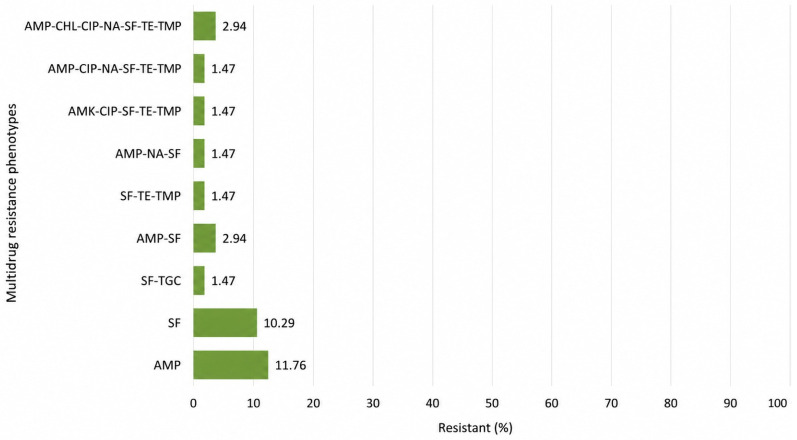
Distribution of phenotypic antimicrobial resistance profiles (resistotypes) among the 68 *E. coli* isolates. Horizontal bars represent the percentage of isolates exhibiting each resistance profile. Profiles were defined according to the combination of antimicrobial agents to which isolates were classified as resistant or non-wild-type (non-WT). Abbreviations: AMK, amikacin; AMP, ampicillin; CHL, chloramphenicol; CIP, ciprofloxacin; NA, nalidixic acid; SF, sulfamethoxazole; TE, tetracycline; TMP, trimethoprim.

Overall, nine different resistance phenotypes were identified among the *E. coli* isolates (Figure 2). The most prevalent phenotypes were resistance to ampicillin alone (11.8%) and sulfamethoxazole alone (10.3%) (Figure 2). Six isolates (8.8%) exhibited resistance to at least one antimicrobial agent in three or more antimicrobial classes and were therefore classified as MDR. Among these, one isolate exhibited resistance to three antimicrobial classes, three isolates showed resistance to five antimicrobial classes and two isolates displayed the most complex resistance profile, involving six antimicrobial classes (Figure 2).

MAR index values ranged from 0.00 to 0.47, with a mean value of 0.046. Ten isolates (14.7%) exhibited MAR values ≥ 0.2, indicating exposure to environments with frequent antimicrobial use. The highest MAR index (0.47) was observed in two isolates displaying resistance to seven antimicrobials belonging to six antimicrobial classes.

**Figure 3:**
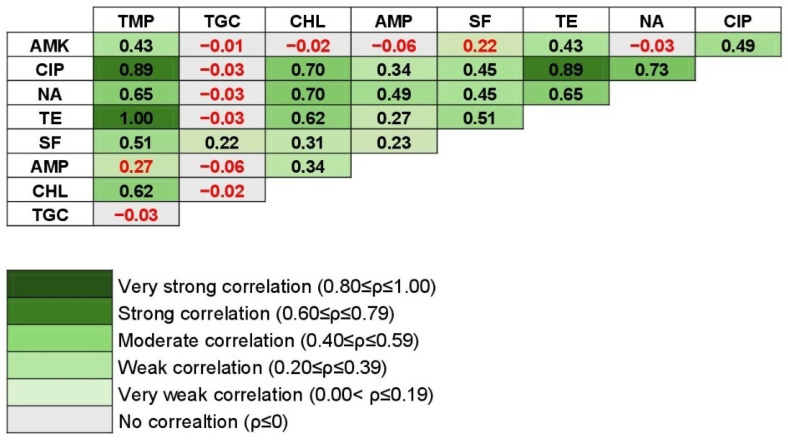
Correlations between antimicrobial resistance phenotypes in *E. coli* isolates based on Spearman’s rank correlation coefficients. Correlation strength was classified as very strong (0.80 ≤ ρ ≤ 1.00), strong (0.60 ≤ ρ ≤ 0.79), moderate (0.40 ≤ ρ ≤ 0.59), weak (0.20 ≤ ρ ≤ 0.39), very weak (0.00 < ρ ≤ 0.19), and no correlation (ρ ≤ 0). Abbreviations: AMK, amikacin; AMP, ampicillin; CHL, chloramphenicol; CIP, ciprofloxacin; NA, nalidixic acid; SF, sulfamethoxazole; TE, tetracycline; TGC, tigecycline; TMP, trimethoprim. Red numbers indicate correlations that were not statistically significant (*p* > 0.05).

Finally, correlations between antimicrobial resistance phenotypes are shown in Figure 3. Notably, very strong positive correlations were observed between resistance to tetracycline and trimethoprim (Spearman’s ρ = 1.00), ciprofloxacin and trimethoprim (ρ = 0.89), and ciprofloxacin and tetracycline (ρ = 0.89) (Figure 3).

None of the six aEPEC isolates exhibited MDR. Three isolates (50%) showed resistance to a single antimicrobial agent (two to ampicillin and one to sulfamethoxazole), whereas the remaining three isolates were susceptible to all antimicrobials tested (Table 2). Overall, no apparent association was observed between the presence of virulence genes and multidrug-resistant phenotypes.

## 4. Discussion

The presence of free-living cats in urban environments has increased considerably in recent decades, leading to the establishment of managed feline colonies in many European countries, including Spain [35]. These colonies are commonly maintained through Trap–Neuter–Return (TNR) programmes aimed at controlling population growth while improving animal welfare. However, feline colonies also pose several challenges, including potential risks to public health, social conflicts, traffic accidents, animal welfare concerns, and negative impacts on local biodiversity [7]. Consequently, appropriate sanitary and veterinary management of these colonies is essential to safeguard both animal and public health. Measures such as suitable colony location, adequate hygiene conditions, population control and regular health monitoring are fundamental components of responsible colony management.

The present study highlights the importance of monitoring AMR, MDR, and virulence-associated traits in stray cats, as these animals may contribute to the environmental dissemination of resistant and potentially pathogenic bacteria, facilitating their transmission to humans and other animals. *E. coli* is widely recognized as a sentinel bacterium for AMR surveillance and is included in several official monitoring programmes involving food-producing animals [36]. Furthermore, certain *E. coli* pathotypes are associated with enteric disease and possess zoonotic potential. As a member of the normal intestinal microbiota of humans and a wide range of animal species, including mammals, birds, and reptiles [37], *E. coli* represents a valuable indicator for investigating both AMR and the circulation of virulence-associated genes within a One Health framework.

In this study, six isolates (8.8%) were classified as atypical enteropathogenic *E. coli* (aEPEC). To the best of our knowledge, studies investigating diarrheagenic *E. coli* pathotypes in feral cats remain scarce, with previous research in these populations focusing primarily on AMR rather than virulence characterization [38]. Although the prevalence detected was relatively low, this finding is epidemiologically relevant because cats have previously been described as asymptomatic carriers and potential reservoirs of diarrheagenic *E. coli* pathotypes, particularly aEPEC [16,17]. The prevalence observed in the present study is consistent with the generally sporadic detection of diarrheagenic *E. coli* in feline populations reported elsewhere [39]. Nevertheless, the identification of aEPEC among free-living cats suggests that these animals may contribute to the environmental persistence and dissemination of potentially zoonotic *E. coli*. Although no clinical signs were observed in the animals included in this study, the detection of aEPEC reinforces the need to include virulence characterization alongside AMR surveillance in feline populations.

None of the aEPEC isolates exhibited MDR. Three isolates showed resistance to a single antimicrobial agent, whereas the remaining three were susceptible to all antimicrobials tested. Although the limited number of aEPEC isolates precludes definitive conclusions, these findings suggest that the virulence-associated traits detected in the present study were not accompanied by extensive AMR. Previous studies conducted in other animal species have reported contrasting results regarding the relationship between virulence and AMR in *E. coli*. While positive associations between specific virulence determinants and AMR have been reported among *E. coli* isolates of ovine origin [40], other authors studying avian *E. coli* have suggested that AMR and virulence-associated genes are not necessarily co-evolved traits [41]. Likewise, reviews on this topic have highlighted that the relationship between virulence and AMR in *E. coli* is complex and may vary according to the host, pathotype, phylogenetic background, and ecological context [42].

In addition to the detection of virulence-associated traits, AMR was also identified among the *E. coli* isolates recovered in this study. Six isolates (8.8%) were classified as MDR. Similar findings have been reported in stray cats from Indonesia [43]. In Europe, relatively few studies have investigated the occurrence of antimicrobial-resistant *E. coli* in free-living cats. Nevertheless, the results obtained in the present study are broadly consistent with those reported in Italy. In Palermo (Sicily), a phenotypic and genotypic characterization of 75 *E. coli* isolates recovered from rectal swabs and faecal samples of stray cats revealed that 43% of the isolates were resistant to at least one of the eight antimicrobials tested, while six isolates (19% of resistant isolates) were classified as MDR [23]. Furthermore, Ratti et al. [22] identified ESBL/AmpC-producing *E. coli* in both pet and stray cats in Italy. Despite the growing interest in AMR in companion animals, epidemiological data from free-living cats remain scarce, limiting direct comparisons among studies. The low mean MAR index (0.046) observed in the present study further supports an overall low level of AMR among the *E. coli* isolates analysed. Nevertheless, 14.7% of the isolates exhibited MAR values ≥ 0.2, indicating that a subset of isolates may have originated from environments subjected to higher antimicrobial selective pressure.

Although data from owned cats should be interpreted with caution, they provide valuable information on the epidemiology of AMR in feline populations. In a study conducted in eastern Spain, 72.6% and 34.7% of *E. coli* isolates from pet cats showed AMR and MDR, respectively [25]. Similarly, higher MDR frequencies have been reported in pet cats from South Korea (20.9%) [44], Zimbabwe (25%) [45], Panama (29%) [46], Hangzhou, China (30%) [47], Bangladesh (46.34%) [48], Thailand (62.1%) [49], and Poland (66.8%) [50]. Collectively, these findings highlight the widespread occurrence of AMR among feline populations worldwide. Therefore, continuous monitoring of *E. coli* antimicrobial susceptibility in companion animals is imperative. Furthermore, the integration and application of recommendations for appropriate use of antimicrobials in small animal practice are essential to minimize the emergence of MDR among *E. coli* in companion animals.

Regarding the antimicrobial agents evaluated, the highest resistance rates were observed for sulfamethoxazole (25.0%) and ampicillin (20.6%). Although both compounds belong to EMA Category D (“Prudence”), the occurrence of resistant isolates may reflect environmental exposure, horizontal transfer of resistance determinants, or indirect selection driven by the use of other antimicrobial agents. Previous studies have demonstrated the widespread occurrence of antimicrobial residues and antimicrobial-resistant bacteria in aquatic and terrestrial ecosystems, highlighting the potential role of the environment in the maintenance and spread of AMR [51,52,53]. Similar resistance patterns, particularly for ampicillin, have previously been reported in both dogs and cats from different geographical regions [25,44]. Resistance frequencies observed for sulfamethoxazole were higher than those reported in some European antimicrobial resistance surveillance programmes [1]. The detection of resistance to these widely used antimicrobial classes is of particular concern because sulphonamides and aminopenicillins remain important therapeutic options in both human and veterinary medicine. These findings are encouraging, as they suggest a limited circulation of resistance mechanisms affecting CIA within the feline colonies studied. Similar findings have not always been reported in studies involving owned cats, where resistance to some of these compounds has occasionally been detected [25,44,54,55].

An encouraging finding of the present study was the absence of resistance to azithromycin, cefotaxime, ceftazidime and colistin. Furthermore, none of the isolates carried the macrolide resistance genes investigated, further supporting the interpretation that azithromycin susceptibility was not associated with acquired resistance mechanisms. Cefotaxime, ceftazidime, and colistin are classified as HPCIAs for human medicine, whereas meropenem is considered a last-resort antibiotic. In addition, very low resistance frequencies were observed for gentamicin, amikacin, chloramphenicol, meropenem and tigecycline. These findings indicate a low prevalence of resistance phenotypes affecting CIA classes and suggest a limited circulation of resistance mechanisms within the feline colonies studied. From a One Health perspective, this observation is particularly relevant because it indicates that resistance to CIA remains uncommon in the population investigated, in contrast to findings reported in some studies involving pet cats, where resistance to these compounds has occasionally been detected [25,54,55].

Overall, the results obtained highlight the need to include free-living cats in antimicrobial resistance surveillance programmes. These animals share urban environments with humans, domestic pets, wildlife, and livestock, creating opportunities for the exchange and dissemination of bacteria, AMR determinants, and virulence-associated traits. Consequently, monitoring both AMR and pathogenic *E. coli* pathotypes circulating among feline colonies should be considered within integrated One Health surveillance strategies.

Several limitations should be considered when interpreting the present findings. First, the sample size was relatively limited and restricted to feline colonies from a specific geographical area, which may limit the extrapolation of the results to other regions. Second, AMR was assessed primarily phenotypically, and a comprehensive molecular characterization of resistance determinants was not performed, although selected macrolide resistance genes were investigated. Likewise, only a limited number of virulence-associated genes were investigated, and other pathogenic *E. coli* pathotypes may therefore have remained undetected. Future studies involving larger sample sizes, broader geographical coverage, and genomic approaches would contribute to a more comprehensive understanding of the epidemiology of AMR, virulence-associated traits, and pathogenic *E. coli* circulating among free-living cats.

## 5. Conclusions

This study provides evidence of AMR and virulence-associated traits in commensal *E. coli* isolated from free-living cat colonies in Spain. Resistance was most frequently observed to sulfamethoxazole (25.0%) and ampicillin (20.6%), whereas all isolates showed high susceptibility rates to most of the antimicrobials tested, including azithromycin (100%), as well as gentamicin, amikacin, cefotaxime, ceftazidime, meropenem, colistin, chloramphenicol and tigecycline (98.5%). Six isolates (8.8%) were classified as multidrug-resistant. In addition, six isolates (8.8%) were identified as atypical enteropathogenic *E. coli* (aEPEC), none of which exhibited multidrug resistance. These findings highlight the importance of including free-living cats in AMR surveillance programmes and support the implementation of integrated One Health monitoring strategies.

## Figures and Tables

**Table 1:** Detection of virulence-associated genes, pathotype classification, and antimicrobial resistance profiles of six atypical enteropathogenic *Escherichia coli* (aEPEC) isolates.

Strain Reference	Gene Detection						Pathotype	Antimicrobial Resistance
	*eae*	*stx1*	*stx2*	*bfpA*	*st*	*lt*		
R18	+	-	-	-	-	-	aEPEC	-
R20	+	-	-	-	-	-	aEPEC	AMP
R22	+	-	-	-	-	-	aEPEC	-
R31	+	-	-	-	-	-	aEPEC	AMP
R74	+	-	-	-	-	-	aEPEC	-
R132	+	-	-	-	-	-	aEPEC	SF

Gene detection legend: *eae*, intimin; *stx1*, Shiga toxin 1; *stx2*, Shiga toxin 2; *bfpA*, bundle-forming pilus subunit A; *st*, heat-stable enterotoxin; *lt*, heat-labile enterotoxin. Isolates positive for *eae* and negative for *bfpA* were classified as atypical enteropathogenic *E. coli* (aEPEC). AMA legend: AMP, ampicillin; SF, sulfamethoxazole.

**Table 2:** Antimicrobial resistance profiles of the 68 *E. coli* isolates.

Antimicrobial Class	Antimicrobial	EMA *	Nº Isolates and Percentage (%)			
			S	%	R	%
**Aminoglycosides**	GEN	C	67	98.5	1	1.5
	AMK	C	67	98.5	1	1.5
**Cephalosporins**	CTX	B	67	98.5	1	1.5
	CAZ	B	67	98.5	1	1.5
**Quinolones**	CIP	B	64	94.1	4	5.9
	NA	B	63	92.6	5	7.4
**Tetracyclines**	TE	D	63	92.6	5	7.4
**Sulfonamides**	SF	D	51	75.0	17	25.0
**Aminopenicillins**	AMP	D	54	79.4	14	20.6
**Carbapenems**	MEM	A	67	98.5	1	1.5
**Macrolides**	AZM	C	68	100.0	0	0.0
**Polymyxins**	CL	B	67	98.5	1	1.5
**Amphenicols**	CHL	C	67	98.5	1	1.5
**Glycylcyclines**	TGC	A	67	98.5	1	1.5
**Diaminopyrimidines**	TMP	D	63	92.6	5	7.4

Abbreviations: GEN, gentamicin; AMK, amikacin; CTX, cefotaxime; CAZ, ceftazidime; CIP, ciprofloxacin; NA, nalidixic acid; TE, tetracycline; SF, sulfamethoxazole; AMP, ampicillin; MEM, meropenem; AZM, azithromycin; CL, colistin; CHL, chloramphenicol; TGC, tigecycline; TMP, trimethoprim. * EMA, European Medicines Agency. According to the EMA classification, Category A = ‘Avoid’; Category B = ‘Restrict’; Category C = ‘Caution’; and Category D = ‘Prudence’. Antimicrobial susceptibility was interpreted according to EUCAST or CLSI clinical breakpoints when available. For antimicrobials lacking clinical breakpoints, epidemiological cut-off values (ECOFFs) were applied. Isolates classified as resistant and non-WT isolates were grouped into a single category.

## Data Availability

The original contributions presented in this study are included in the article/supplementary material. Further inquiries can be directed to the corresponding author.

## Supplementary Materials

The following supporting information can be downloaded at https://www.mdpi.com/article/10.3390/ani16152435/s1. Table S1. Primer sets for macrolide resistance targets. Table S2. PCR oligonucleotides sequences used for pathotyping determination, amplicon sizes and PCR conditions used for amplification.
