# Supplementary material for: Antimicrobial Resistance and Selected Virulence-Associated Genes Escherichia coli Pathotypes in Free-Living Cats from Southern Spain
Source: Animals (Basel). 2026 Aug 6;16(15):2435. doi: 10.3390/ani16152435 (PMC13464435; doi:10.3390/ani16152435)
Supplement: Supplementary file 1 [file animals-16-02435-s001.zip › animals-4411500-supplementary.pdf]

S1 Table. Primer sets for macrolide resistance targets

Target		Primers		Size (bp)	Ann (ºC)	Ref.
Gene	Prot	Forward (5’-3’)	Reverse (5’-3’)			
Macrolide Resistance Genes						
ere(A)	EreA	GCCGGTGCTCATGAACTTGAG	CGACTCTATTCGATCAGAGGC	420	60	Gomes et al., 2019
erm(A)	ErmA	TCTAAAAAGCATGTAAAAGAAA	CGATACTTTTTGTAGTCCTTC	533	52	Gomes et al., 2019
erm(B)	ErmB	GAAAAAGTACTCAACCAAATA	AGTAACGGTACTTAAATT	639	45	Gomes et al., 2019
erm(C)	ErmC	TCAAAACATAATATAGATAAA	GCTAATATTGTTTAAATCGTCAAT	642	45	Gomes et al., 2019
mef(A)	MefA	AGTATCATTAATCACTAGTGC	TTCTTCTGGTACTAAAAGTGG	345	54	Gomes et al., 2019
mef(B)	MefB	ATGAACAGAATAAAAAATTG	AAATTATCATCAACCCGGTC	1255	45	Gomes et al., 2019
mph(A)	MphA	GTGAGGAGGAGCTTCGCGAG	TGCCGCAGGACTCGGAGGTC	403	60	Gomes et al., 2019
mph(B)	MphB	ATTAAACAAGTAATCGAGATAGC	TTTGCCATCTGCTCATATTCC	868	50	Gomes et al., 2019
msr(A)	MsrA	GCACTTATTGGGGGTAATGG	GTCTATAAGTGCTCTATCGTG	384	58	Gomes et al., 2019
msr(D)	MsrD	CCCCAGTTGGACGAAGTAA	TTGTTTTTCCGATTCCATTAC	781	50	Gomes et al., 2019
Macrolide Chromosomal Targets						
rplD	L4	GGCAAGAAAATGGCAGGTCAGATGG	TTCCATCGCAGTAGACGCTTTTTCA	846	56	Gomes et al., 2019
rplV	L22	CGGTGGAAAGCGGAGACAAGAAGCC	ACCAGTTTTGCGTCCAGTTCAGGCT	926	56	
rrlH[a]	23S	TAAGGTAGCGAAATTCCTTGTCG	TGATGCGTCCACTCCGGTC	756	61	

S2 Table. PCR oligonucleotides sequences used for pathotyping determination, amplicon sizes and PCR conditions used for amplification

Primer code	Oligonucleotide sequence 5' to 3'	Target gene	Pathogenic *E. coli* strain	PCR conditions						Product size (Bp)	Reference
				**Denaturation**		**Annealing**		**Extension**			
				Temp	Time	Temp	Time	Temp	Time		
EAE1<br>EAE2	F: AAACAGGTGAAACTGTTGCC<br>R: CTCTGCAGATTAACCTCTGC	*eae*	EPEC/EHEC	95ºC	40 sec	55^0C	30 sec	72ºC	1 min	490	Belete et al., 2022
EVS1<br>EVC2	F: ATCAGTCGTCACTCACTGGT<br>R: CTGCTGTCACAGTGACAAA	*stx1*	STEC/EHEC	95ºC	40 sec	55^0C	30 sec	72ºC	1 min	110	Belete et al., 2022
EVT1<br>EVT2	F: CAACACTGGATGATCTCAGC<br>R: CCCCCTCAACTGCTAATA	*stx2*	STEC/EHEC	95ºC	40 sec	55^0C	30 sec	72ºC	1 min	350	Belete et al., 2022
BFPF<br>BFPR	F: AATGGTGCTTGCGCTTGCTGC<br>R: GCCGCTTTATCCAACCTGGTA	*bfpA*	Typical EPEC	95ºC	40 sec	45^0C	40 sec	72ºC	1 min	324	Belete et al., 2022
ST1<br>ST2	F: TTTATTTCTGTATTGTCTT<br>R: GCAGGATTACAACACAATTC	*st*	ETEC	95ºC	40 sec	55^0C	30 sec	72ºC	1 min	294	Belete et al., 2022
LT1<br>LT2	F: GCTCTATGTGCATACCGAGT<br>R: CCATACTTGATTGCCGCAAT	*lt*	ETEC	95ºC	40 sec	48^0C	30 sec	72ºC	1 min	696	Belete et al., 2022

Temp= Temperature, Sec= Second, Min= Minute
